# Mammography and MRI manifestations of breast angiosarcoma

**DOI:** 10.1186/s12905-019-0769-3

**Published:** 2019-06-10

**Authors:** Wen-Hai Wu, Qing-Lin Ji, Zhi-Zheng Li, Qian-Nan Wang, Shu-Ying Liu, Jin-Fen Yu

**Affiliations:** 1 Department of Radiology, JiNan ZhangQiu maternal and child health hospital, ShanDong, 250200 China; 2 Magnetic Resonance Room, JiNan ZhangQiu District Hospital of TCM, ShanDong, 250200 China

**Keywords:** Breast Angiosarcoma, Mammography, MRI

## Abstract

**Background:**

Breast angiosarcoma is rare and previous studies only focus on its pathology. This study aimed to summarize its imaging features.

**Methods:**

Overall 17 patients pathologically confirmed with breast angiosarcoma were recruited. Eight patients underwent preoperative mammography, and 13 received preoperative MRI scan. The mammography and MRI findings were classified according to the ACR-BI-RADS-mammography/MR lexicon.

**Results:**

Mammography showed that 3 cases developed diffuse asymmetry occupying two or more quadrants of the affected breast and that 5 patients had lobulated or oval masses. The 13 patients’ lesions presented as diffuse and slightly/significantly high homogeneous or heterogeneous signals on T1-weighted images, while the necrotic and cystic parts had relatively low signals. The hemorrhagic lesions in 7 cases had high signals on both T1- and T2-weighted images. A hemosiderin ring at the edge of an old hemorrhagic lesion had long and short signals on the T1- and T2-weighted images, respectively. Contrast-enhanced MRI revealed that the 13 patients’ lesions had significant heterogeneous enhancement. Significant enhancement was observed in the early phase, and varying degrees of concentric enhancement was seen in the delayed phase.

**Conclusions:**

The mammography findings are nonspecific. MRI scan is helpful in determining the malignancy of the lesions. Breast angiosarcoma usually shows heterogeneous signals on both T1-weighted and T2-weighted images. Due to their incomplete lumens and lack of thrombocytes, patients with angiosarcoma have a higher incidence of bleeding (nearly 50% in this study) than those with other malignant tumors. The pattern of the enhancement curve helps to distinguish this disease from the typical types of breast cancer.

## Background

Breast angiosarcoma, accounting for approximately 8% of breast sarcomas and 0.04% of all breast malignancies [1], is a highly malignant tumor of spindle cells. There are primary breast angiosarcoma (PBA) and secondary breast angiosarcoma (SBA) [2]. PBA usually occurs to young women without any inducing factor, while SBA is commonly seen in older women who have a history of breast surgery and radiotherapy. PBA is rarely seen in clinical practice and it is predicted that PBA will have a higher morbidity and be more aggressive in the future [3–5]. Increasing use of breast-conserving surgery leads to increase in the incidence of SBA and among all secondary tumors resulted from radiotherapy, SBA is believed to be the most common one. Since breast angiosarcoma is extremely rare, previous literature is only about case reports on the pathology of this disease, and no study of imaging findings has been reported. In this study, a comprehensive analysis of 17 patients’ clinical reports and imaging findings was done to improve understanding and recognition of the clinical and imaging features of breast angiosarcoma.

## Methods

### Clinical data

We collected the clinical and imaging data of 17 female patients with breast angiosarcoma (15 with PBA and 2 with SBA) which was pathologically confirmed in five hospitals (including our hospital) from April 2005 to June 2018. The 17 patients’ mean age was 34.6 years (range: 17–48). Among the 15 patients with PBA, 14 did not have any precursor disease and no similar lesions were found on the other body parts, while the other 1 had bruises on the skin of her affected breast. The bruises were caused by a hard object one year before the study. The injured skin became dark, swollen and indurated, and anti-inflammatory treatment did not improve the condition. The two cases of SBA developed this disease secondarily to angiosarcoma of other body parts (the cervical vertebra and the liver, respectively).

The patients had PBA/SBA 2 to 16 months ago, and the masses became progressively more swollen. Four patients suffered severe breast pain. Five cases had purplish-red breast skin, and three of them experienced edema of the affected breast. No skin ulcers or varicose veins were found in any patients. The results of surgical pathological examination showed that the average maximum diameter of the tumors was 8.6 cm (range: 4–16.5).

### Diagnosing methods

Eight patients underwent preoperative mammography in a standing position with GE Digital Senographe 2000D Mammography (GE Healthcare, Waukesha, Wisconsin) and Siemens Mammomat Novation DR (Siemens Healthcare, Erlangen, Germany) under automatic exposure mode. Both the craniocaudal (CC) and mediolateral oblique (MLO) views were examined. Thirteen patients underwent MRI scan using GE Signa HDx 3.0 T MRI machine with dedicated breast surface coils. Non-enhanced imaging: T1WI: TR/TE = 800/8 ms, STIR: TR/TE = 3300/85 ms, the slice thickness = 6 mm, the matrix = 320 × 192; dynamic contrast-enhanced gradient-echo imaging: T1WI: TR/TE = 4.2/2.1 ms, the flip angle = 10 °, the slice thickness = 4 mm, the matrix = 384 × 256, the NEX = 1, the FOV = 30 × 38 cm^2^. Gd-DTPA of 0.1 mmol/kg was injected into the median cubital vein as the contrast agent. Enhanced scan was done before the injection of Gd-DTPA and 1, 2, 4, and 7 min after it.

### Analysis of images

Necrosis, cystic degeneration and hemorrhagic regions were avoided to be included in the enhancement curves. The morphology, diameter, components, and margins of the tumors, skin changes and the condition of the axillary lymph nodes were analyzed retrospectively by two radiologists who had practiced imaging diagnosis of breast diseases for more than five years. They had no access to patients’ previous clinical records and reached a consensus through discussion when they had different opinions.

### Pathological analysis

The samples were fixed, decalcificated, stained with hematoxylin-eosin (HE), and then examined with optical microscope. Classification of the samples was done according to immunohistochemistry results. The sections were examined by two senior professors who had practiced pathological diagnosis of breast tumors for more than 15 years.

## Results

### Mammography

**Fig. 1:**
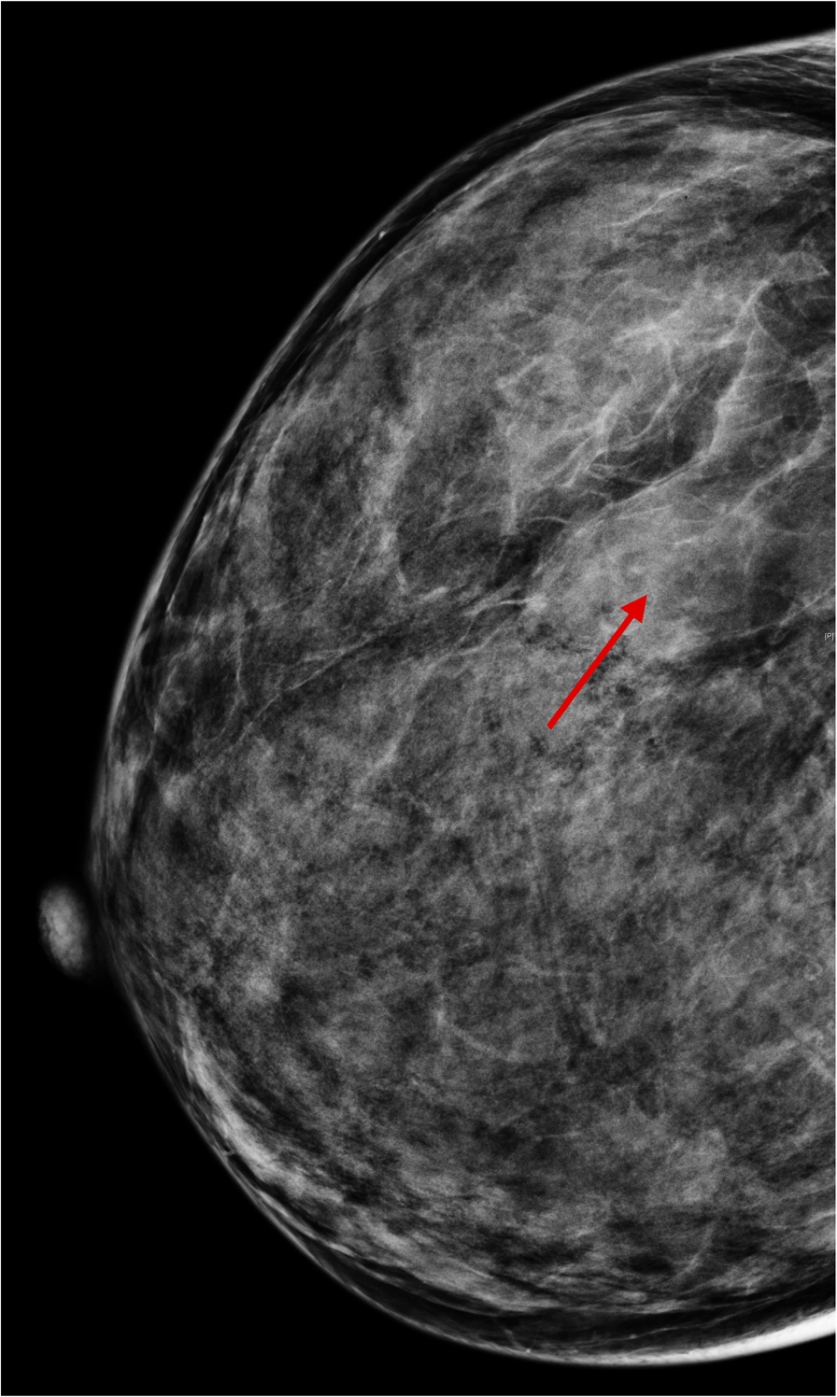
A female whose right breast has been swollen for 2 months. There is a solitary mass in the upper outer quadrant of her right breast (red arrow). The mass is well-defined, though part of the borders is obscured. The margins are not smooth. No calcification or thickening of the skin is observed

Among the 8 patients who underwent mammography (Table 1), 5 cases presented with deep-seated lobular or oval masses (Fig. 1). Three patients’ mammograms showed huge asymmetrical mass shadow that occupied two or more quadrants and fused together with linear low-density parts, poorly marginated lesions, and trabecular thickening of surrounding tissues (Fig. 4a). The margins of masses were not smooth, and some of them were even obscured. Skin edema was found in 2 cases. All the 8 cases did not experience calcification or swollen axillary lymph nodes.

**Table 1:** The clinical and mammography features in breast angiosarcoma

Case N0.	Age range (years)	Site of breast disease	Size of mass (cm)	Skin alterations	Mammography findings	Mass boundary
1	40–50	Right	15 × 9.8	Edema, amaranth	Lobulated mass	Rough
2	40–50	Left	4.1 × 3.8	None	Oval mass	Rough
3	20–30	Left	6 × 4.2	None	Lobulated mass	Shelter
4	50–60	Left	4.8 × 3.7	None	Lobulated mass	Shelter
5	40–50	Right	8 × 7.2	Black	Diffuse asymmetry	None
6	30–40	Right	5.3 × 6.8	Edema, amaranth	Oval mass	Rough
7	30–40	Right	4.5 × 6.0	None	Diffuse asymmetry	None
8	40–50	Right	14.3 × 16.5	None	Diffuse asymmetry	None

### MRI scan

**Fig. 2:**
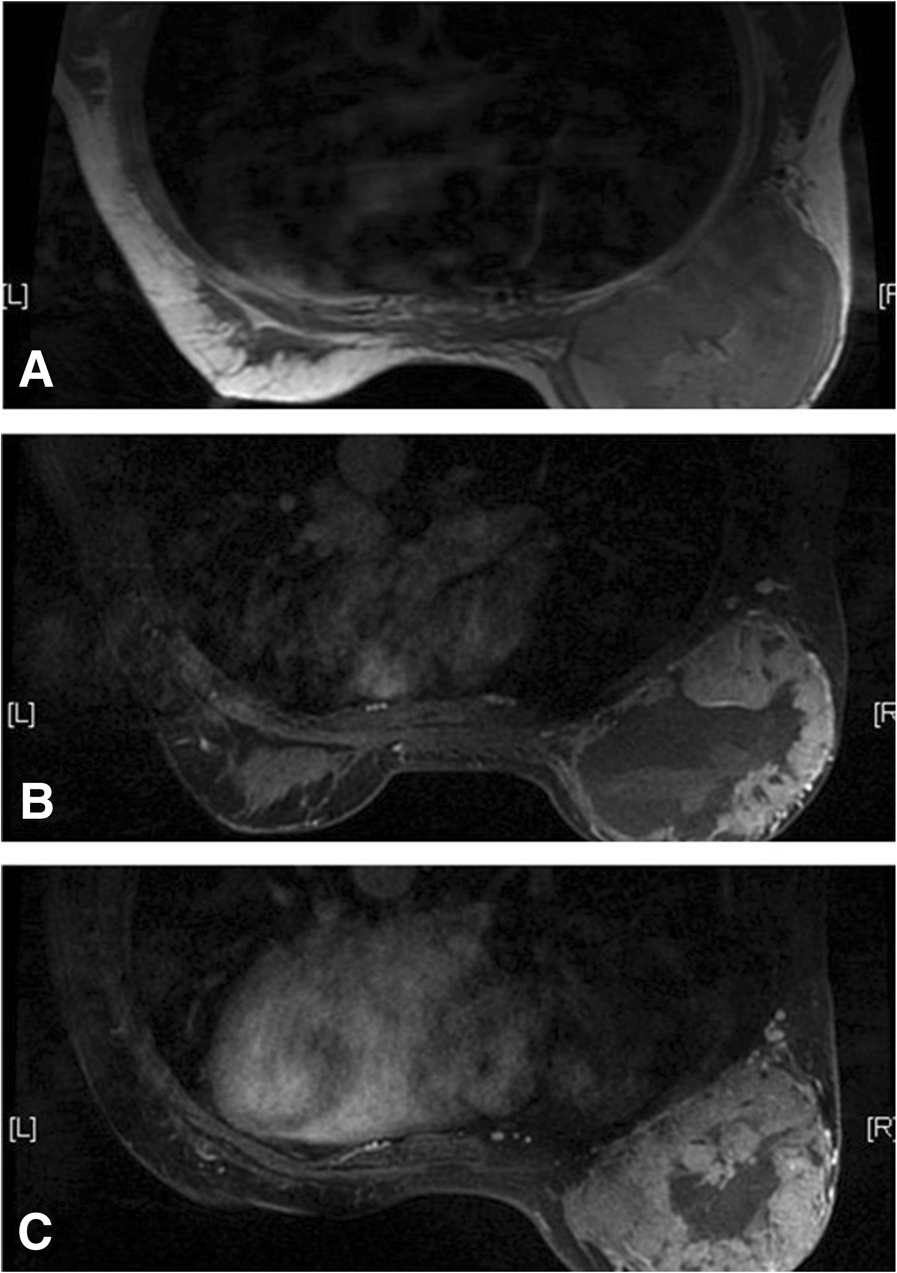
A female whose right breast has been swollen for 1 months. The mass has been enlarging in a high rate since she was pregnant recently. **a**: On T1-weighted images, the hemorrhagic lesion shows equal and high signal intensity. **b**: The contrast enhanced images demonstrate significant and heterogeneous enhancement in the early stages. **c**: Concentric enhancement of the contrast agent is observed in the delayed phase

**Fig. 3:**
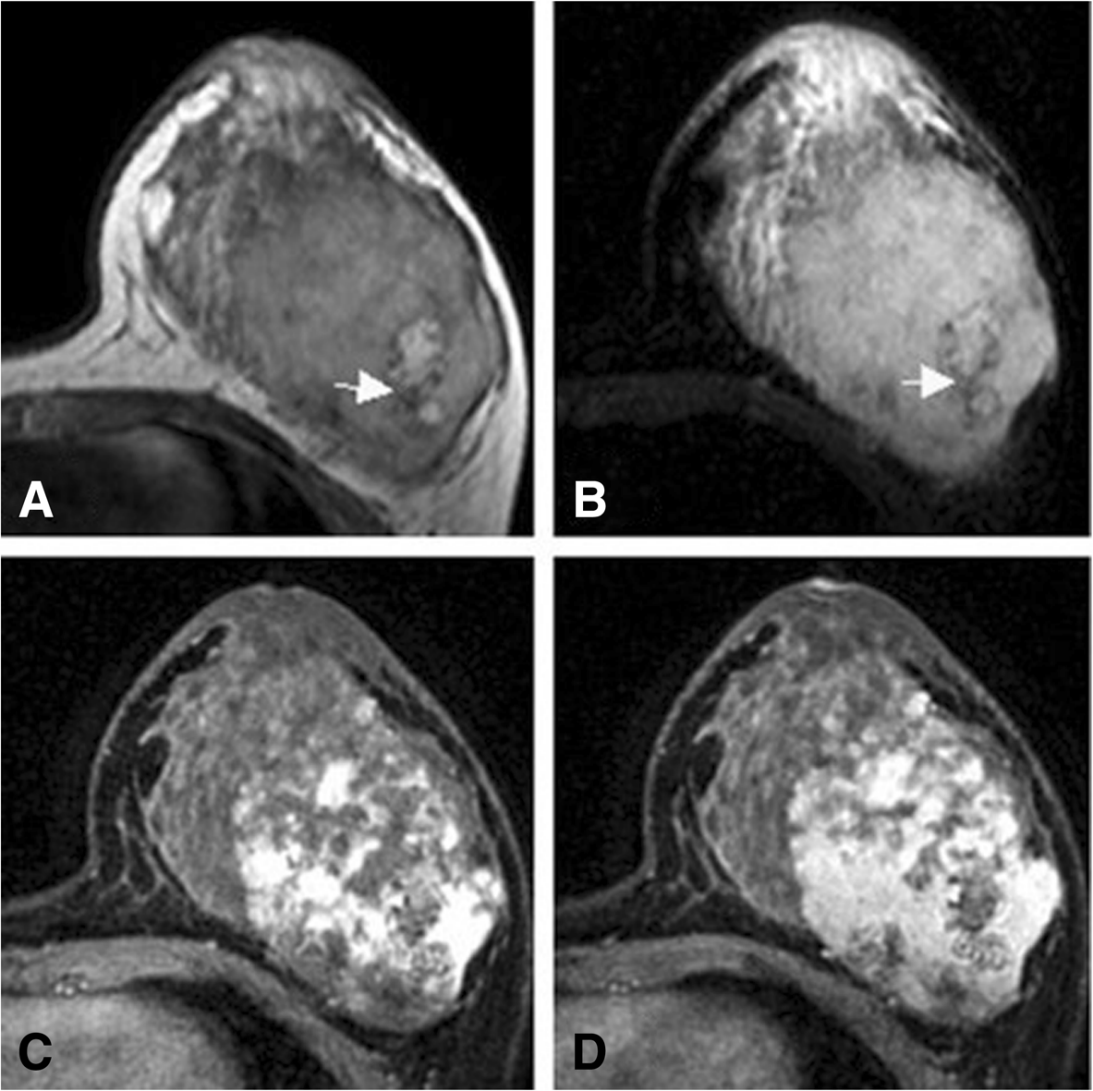
A female who had a mass in her left breast for 16 months. The mass has been enlarging in a high rate for three months since she was pregnant recently. The hemosiderin ring at the edge of the hemorrhagic lesion has long T1 and short T2 signals (white arrow). **a**: On T1-weighted images, the lesion shows heterogeneous signal intensity. **b**: On fat-suppressed T2-weighted images, the lesion shows bright (high) signal intensity. **c**: The contrast enhanced images demonstrate heterogeneous enhancement in the early stages. **d**: Concentric enhancement is observed in the delayed phase

**Fig. 4:**
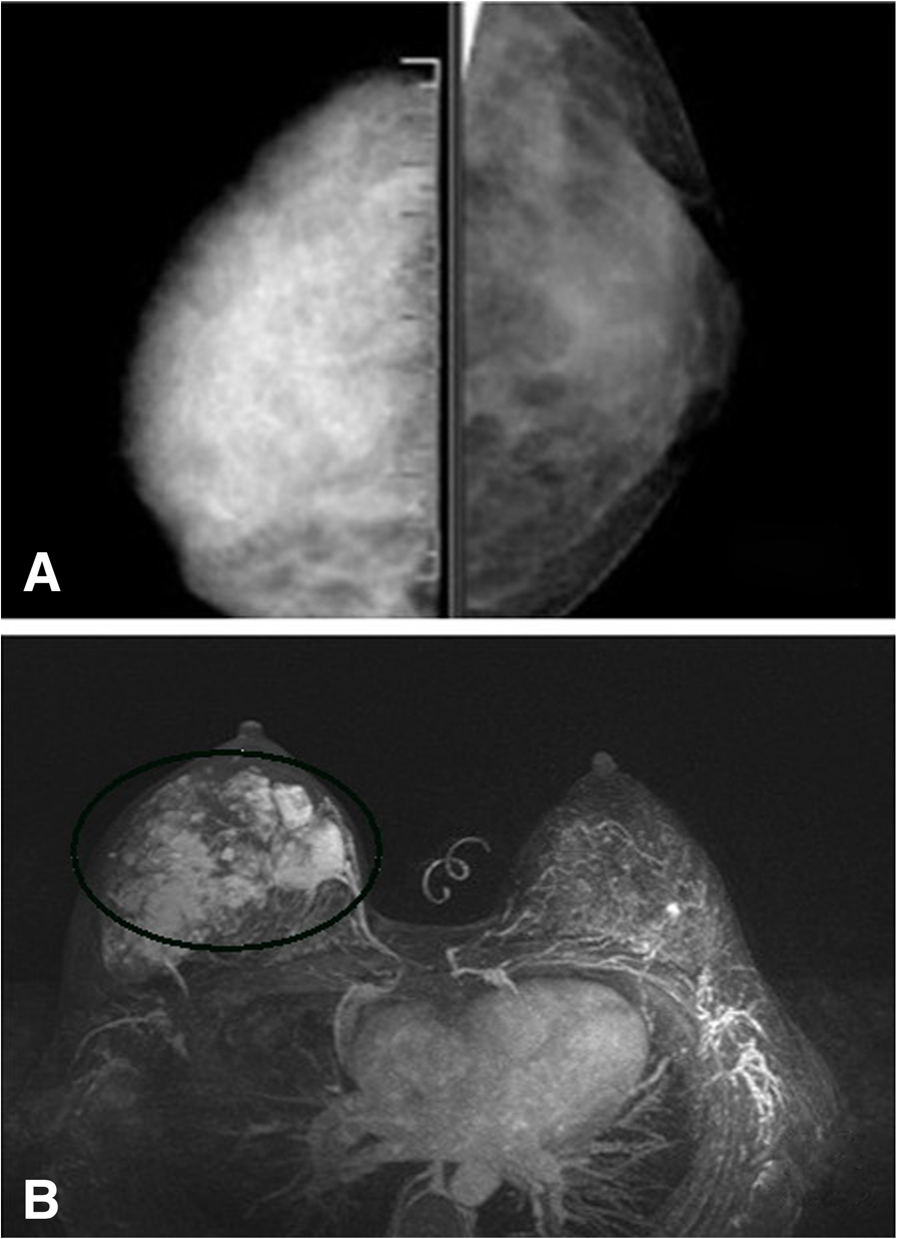
A female whose right breast has been swollen for 11 months. **a**: Diffuse dense shadow is observed in the craniocaudal view of the right breast. **b**: The contrast enhanced MR images reveal multiple heterogeneous enhanced masses

**Fig. 5:**
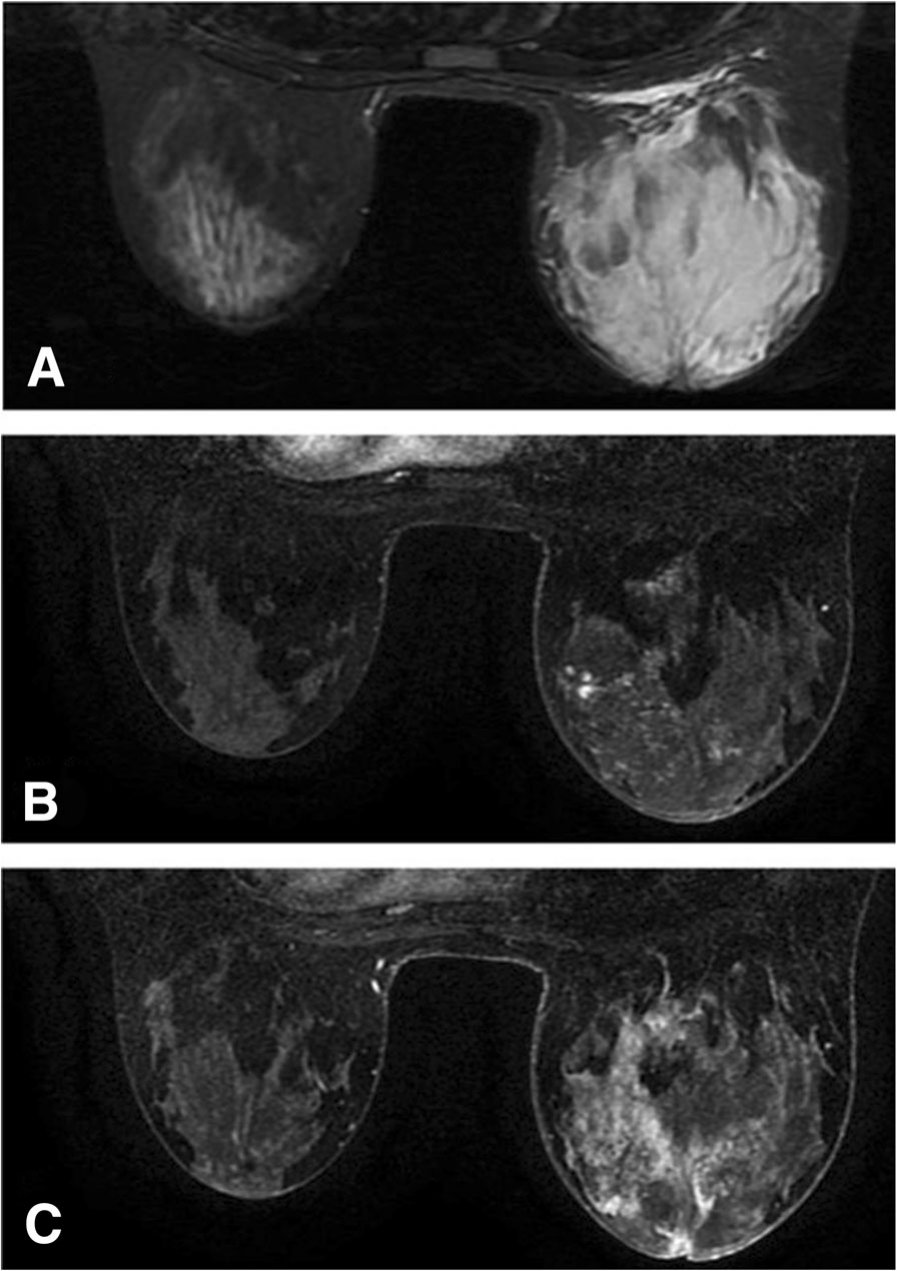
A female who has had a mass in her right breast for 7 months. **a**: On T2- weighted images, the mass shows bright (high) signal intensity; **b**: The contrast enhanced images reveal non-mass-like enhancement in the early stages; **c**: Partial concentric enhancement of the contrast agent is observed in the delayed phase

Thirteen patients received preoperative MRI scan (Table 2). Lesions had slightly/significantly high homogeneous or heterogeneous signals on T1-weighted images (Fig. 2a, 3a). Necrosis and cystic degeneration exhibited relatively low signals. Hemorrhagic lesions were found in 7 cases and had high signals on T1-weighted and T2-weighted images (Fig. 2a, 3a, b). The hemosiderin ring resulted from long-term hemorrhage had long and short signals on T1- and T2-weighted images, respectively (Fig. 3a, b: white arrow). Three patients suffered from edema, thickening, and cloudy subcutaneous fat of the breast skin. Contrast-enhanced images revealed significant heterogeneous enhancement of all lesions (Figs. 2, 3, 4 and 5). Significant enhancement was observed in the early phases, and concentric enhancement at different degrees was seen in the delayed phase (Figs. 2, 3). The dynamic contrast enhancement curves of most cases were in a persistently enhancing or plateau pattern, while the curve of one patient was in a washout pattern. One patient’s lesions were enhanced in a non-mass-like form, and had no well-defined margins (Fig. 5). The patient’s dynamic contrast enhancement curve was in a persistently enhancing pattern.

**Table 2:** The MRI features of breast angiosarcoma

Case No.	Age range (years)	Site of breast disease	Size (cm)	Mass boundary	Signal on T1WI	Signal on T2WI	Bleeding	Enhancement
1	30–40	Left	4 × 3.5	Irregular	Low	High	No	Centripetal heterogeneous filling
2	10–20	Left	4 × 7	Regular	Uneven low	Uneven high	No	Centripetal heterogeneous filling
3	40–50	Left	7 × 8	Irregular	Uneven low	Uneven high	Yes	centripetal heterogeneous filling
4	40–50	Right	7 × 8.2	Irregular	Uneven low	Uneven high	Yes	Rim
5	30–40	Right	5.3 × 6.8	Irregular	Uneven low	Uneven high	Yes	Centripetal heterogeneous filling
6	30–40	Right	4.5 × 6.0	Regular	Uneven low	Uneven high	No	Centripetal heterogeneous filling
7	40–50	Right	16.5 × 14.3	Regular	Uneven low	Uneven high	Yes	Centripetal heterogeneous filling
8	50–60	Right	8.3 × 5.0	Regular	Uneven low	Uneven high	Yes	Heterogeneous
9	20–30	Left	7.0 × 8.0	Regular	Uneven low	Uneven high	Yes	Centripetal heterogeneous filling
10	20–30	Right	4.7 × 6.3	regular	Low	Uneven high	No	Rim
11	30–40	Right	5.7 × 7.4	irregular	Uneven low	Uneven high	No	Heterogeneous
12	30–40	Left	8.8 × 9.5	irregular	Uneven low	Uneven high	Yes	Heterogeneous
13	30–40	Right	5.9 × 8.3	irregular	Uneven low	Uneven high	No	Centripetal heterogeneous filling

### Pathological analysis

All the lesions of the 17 cases were located in the breast parenchyma. These soft lesions with fish-meat- or sponge-like cross sections were rich in blood, and had no capsules and well-defined margins. However, different regions had different characteristics and the characteristics also varied with the differentiation degree of tumor tissues. Lesions were characterized by irregular capillary lumens. The poorer the differentiation was, the more common atypia and nuclear division in vascular endothelial cells were. Blood lakes resulted from excessive hemorrhage were seen, while the vascular compartment and breast ducts were hardly observed.

### Follow-up

The 17 patients’ pathological sections showed no metastasis to axillary lymph nodes. The patients received modified radical mastectomy or simple mastectomy, followed by adjuvant radiotherapy and chemotherapy. Follow-up was conducted with 12 patients. One case was found to have metastatic tumors in multiple vertebral bodies and the pelvis while receiving a bone scan 14 months after surgery. Abdominal CT scan indicated no abnormalities, and breast ultrasound revealed no local recurrence. The patient then received nine courses of chemotherapy. Bone scan, abdominal CT scan and mammography done 25 months after surgery indicated no new lesions. Pulmonary metastasis was observed in 1 case 7 months after surgery, while metastases to the cervical spine and lung was found in another patient 19 months after surgery. Three patients died 25 to 41 months after surgery (2 from multiple organ failure). No recurrence or distant metastasis was found in the other six patients who had been followed up for 9 to 21 months. Five patients were lost to follow-up and they could not be contacted by telephone.

## Discussion

Angiosarcoma, also known as malignant hemangioendothelioma, is derived usually from the breast parenchyma and occasionally from the skin, accounting for less than 0.04% of all malignant breast cancers [6–9]. It occurs in both the primary and secondary forms. The first case of breast angiosarcoma was reported by Borrman in 1907, and the first case of SBA was reported by Body in 1987 [9]. Increasing use of whole breast irradiation after breast-conserving surgery has led to a much higher incidence of secondary angiosarcoma than that of primary angiosarcoma. It was reported that the median age of onset of PBA ranged from 30 to 50 years, while that of SBA was 40 to 60 years [9]. In this study, the age of onset is relatively younger: the average age of onset is 34.6 years, and the youngest patient is only 17 years old, indicating a tendency of breast angiosarcoma’s affecting of younger people.

Adjuvant radiotherapy is a major risk factor for SBA, accounting for 1% of this tumor [9]. Most cases of SBA occur in the surrounding area of the irradiation region and may be associated with chronic lymphatic edema. In the present study, 14 cases of PBA have no precursors, while this disease occurs in another PBA patient after a breast injury. The 2 cases of SBA are secondary to angiosarcomas of other sites.

Microscopic examination indicates that PBA is derived from the mesenchyme, while angiosarcoma cells caused by radiotherapy and other factors often gather in the dermis [10]. Microscopic examination reveals that the 15 cases of PBA are derived from the mesenchyme. Though one of the 15 patients developed PBA after her breast was injured, imaging and pathological analysis results demonstrate that no skin cells are involved. Thus, we consider that this case is still a case of PBA, and that the injury is not the direct cause of angiosarcoma.

PBA may present as palpable masses and enlarged breast. Masses may occupy the breast fully, and some of them may progress in a high speed. Patients with SBA present with multiple painless lesions, and sometimes with masses [11]. Other signs such as bluish or purplish discoloration indicate vascular tumors. Two cases in this study experienced diffuse swelling, pain, redness, blackish discoloration and edema of the affected breast, which is similar to the symptoms of mastitis. Tumor growth in two cases accelerated after the patients were pregnant.

Overall, breast angiosarcoma has a poor prognosis with a 5-year survival rate of approximately 33% [11]. Hematogenous metastasis is a major way for angiosarcoma to spread to other body parts, while a small group of patients develop lymphatic metastasis. The lungs are reported to be the most common site of metastasis [12]. However, many cases are reported to experience gastrointestinal bleeding resulted from the metastasis of angiosarcoma to the cecum [13]. In this study, one patient developed multiple bone metastases 14 months after surgery. Currently, aggressive treatment of breast angiosarcoma is necessary, however, the efficacy of neoadjuvant chemotherapy, adjuvant chemotherapy, and radiotherapy remains unknown.

The mammography findings of breast angiosarcoma are nonspecific. Angiosarcoma can be a solitary well- or ill-defined mass. Normally, it is not associated with calcification or spiculation. According to the mammography results of this study, PBA can present as well-defined round nodules, a large asymmetric dense shadow that affects the whole breast with uneven density, breast trabecular disorder, thickened local vessels, cloudy subcutaneous fat, and skin thickening. No enlarged axillary lymph nodes are observed. These findings are in conformity with the features of sarcoma metastasis via blood vessels. No calcification is seen in patients with PBA. One possible explanation can be that this disease seldom affects the breast parenchyma, which leads to no intra-ductal or intra-lobular comedo necrosis, or secondary calcium deposition. Besides, because the lesions are highly malignant and develop in a high rate, hyaline degeneration and secondary coarse calcification rarely occur in the breast mesenchyme.

Numerous studies have reported the potency of MRI in determining the malignancy of angiosarcoma. Angiosarcoma exhibits high signal intensity on T2-weighted images; significant enhancement is observed in the early stages; and the enhancement curve is in a washout pattern [14]. Large masses have high signal intensity on both the T1-weighted and T2-weighted images, causing thrombocytopenia and secondary bleeding [15]. The 13 patients’ preoperative MRI scan reveals that angiosarcoma can be a mass with three-dimensional features and well-defined borders. All the signals inside the mass are heterogeneous. Necrosis, cystic degeneration, and hemorrhage may occur. A hemosiderin ring is observed at the edge of a long-term hemorrhagic lesion. Our results also demonstrate that angiosarcoma can also be a non-mass-like enhanced multifocal and diffuse lesion with two-dimensional features and ill-defined borders. Most of the signals inside the lesion is the same as those of the normal glandular tissues.

Though significant enhancement of lesions in 13 cases is observed in the early phases, the enhancement is progressive and concentric. In addition, enhancement is observed in the central region of the lesions in 8 cases. All the three patterns of the dynamic enhancement curves are observed in the 13 cases, however, the persistently enhancing pattern and the plateau one are two main types (10/13). The pattern of the dynamic enhancement curve is associated with the microscopic findings of angiosarcoma. A well-differentiated angiosarcoma is rich in capillary networks and the lumens are complete. It takes a longer period for the contrast agent to pass throughout the tumor and to be washed out. Therefore, the curve is in a persistently enhancing or a plateau pattern. The major components of a poorly differentiated tumor are scattered malignant vascular endothelial cells. Lack of capillary networks and incomplete lumens contribute to easy and rapid washout of the contrast agent.

### Differential diagnosis

Breast angiosarcoma presenting as masses or asymmetry dense lesions should be firstly differentiated from breast cancer which has various forms. The median age of onset for breast cancer (more than 40 to 50 years) is older than that for breast angiosarcoma. Breast angiosarcoma should be further differentiated from breast inflammation. The two kinds of diseases share several common things in the clinical symptoms, age of onset, and imaging manifestations, which makes differentiation difficult. However, breast angiosarcoma cannot be relieved by anti-inflammatory treatment. Breast angiosarcoma should also be differentiated from breast lymphoma. The imaging manifestations of breast lymphoma are nonspecific and the age of onset for this disease is similar to that for breast angiosarcoma. Patients with breast lymphoma have a higher incidence to present with swollen axillary lymph nodes, which can help the differentiation between the two breast diseases. Besides, a breast angiosarcoma presenting as a solitary mass should be also differentiated from phyllodes tumor.

## Conclusions

Mammography and MRI manifestations indicate that breast angiosarcoma may present in various forms, uneven signals and heterogeneous enhancement. There are six points which can be helpful for the diagnosis of this tumor.

① Women who age 20 to 40 years old are most likely to develop breast angiosarcoma, and some teens also suffer from this tumor.
② Little calcification is observed within the lesions.
③ Though patients’ history of this tumor is short, the whole breast is usually involved. Swollen axillary lymph nodes are rarely seen.
④ The incidence of skin edema and redness is relatively high and the two symptoms cannot be easily relieved with anti-inflammatory treatment. Purplish-blue discoloration of the skin is a highly effective indicator of the origin of vessels.
⑤ On T1-weighted images, except for the necrotic and cystic parts, the other parts of the lesions have equal or relatively higher signal intensity as the major signal intensity, which is different from the slightly lower signal intensity on T1-weighted images of breast cancer or inflammation. The signals of the solid parts are occasionally higher than those of the necrotic or cystic regions on T2-weighted images. Due to incomplete lumens and lack of thrombocytes, patients with angiosarcoma have a higher rate of bleeding (nearly 50% in this study) than those with other malignant tumors.
⑥ Although the morphology of breast angiosarcoma is similar to that of typical malignant tumors, it takes a longer period for the dynamic enhancement curve of the former to reach a washout pattern**.** Besides, enhancement can be observed in the delayed phase.

## Data Availability

Data to replicate findings are in the Figures and Tables of the main paper. Due to patient privacy protection, any additional materials of the study are only available upon individual request directed to the corresponding author.

## Abbreviations

MRI: magnetic resonance imaging
PBA: primary breast angiosarcoma
SBA: secondary breast angiosarcoma
